# Britain Breathing: using the experience sampling method to collect the seasonal allergy symptoms of a country

**DOI:** 10.1093/jamia/ocx148

**Published:** 2017-12-12

**Authors:** Markel Vigo, Lamiece Hassan, William Vance, Caroline Jay, Andrew Brass, Sheena Cruickshank

**Affiliations:** 1 School of Computer Science, University of Manchester, Manchester, UK; 2 Health eResearch Centre, Farr Institute for Health Informatics Research, Manchester, UK; 3 School of Biological Sciences, University of Manchester, Manchester, UK

**Keywords:** ecological momentary assessment, mobile applications, rhinitis, allergic, seasonal

## Abstract

**Objective:**

Allergies are increasing, but the reasons for this are unclear. Although environmental factors are thought to be important, there is a lack of data on how they contribute to symptom development. To understand this relationship better, we need accurate data about both symptoms and environmental factors. Our objective here is to ascertain whether experience sampling is a reliable approach for collecting allergy symptom data in the general population, allowing us to map symptoms and understand etiology.

**Materials and Methods:**

We conducted a 32-week cross-sectional study where individuals reported their seasonal allergy symptoms and severity via a mobile application. Symptom geographical location and timestamp were also collected automatically.

**Results:**

The experience sampling method reliably infers the incidence of seasonal allergies as indicated by the strong correlation (*r* = 0.93, *P* < .003) between the reported lack of wellness and the number of antihistamines prescribed by General Practitioners.

**Discussion and Conclusion:**

The project has resulted in the first dataset to map allergy symptoms over time and place and reveals periods of peak hay fever symptoms in the UK.

## INTRODUCTION

We are witnessing a dramatic rise in the incidence of seasonal allergies and asthma. Reports reveal that the prevalence of these conditions is 10%–30% of the population, with especially high incidence in the developed world, and some reports suggest that as many as 40% of children in the UK have allergic rhinitis.^1^ The reasons for this rise in allergies are unclear, but links between increased hygiene, reduced early exposure to infection, and increased exposure to pollutants have all been suggested.^2^ It is anticipated that warmer temperatures and higher pollution levels could make allergies even more common,^3^ with young individuals and those living in developing countries being most vulnerable. Although primary care records provide good data in terms of diagnosis, drug prescriptions, and hospital admissions for severe flares of allergy, there is a paucity of data tracking the ebb and flow of symptoms over time. Importantly, no dataset has yet captured accurate information about the time and location of reported symptoms, which could then be used to map the incidence of allergy in the general population to, for example, infer linked environmental factors.

The ultimate aim of the Britain Breathing project is to identify environmental factors that may cause or exacerbate allergy symptoms.^4^ While datasets containing geolocated time series of pollen count and pollution are becoming widely available,^5^^,^^6^ there is no equivalent for seasonal allergy symptoms. One approach to gathering allergy incidence is to mine social media; for instance, Twitter has been used by epidemiologists to monitor H1N1 outbreaks.^7^ A major limitation of this approach is a lack of geographical information. Only 26% of Twitter users include location data in posts,^8^ and when location is shared, it is done at a city level, which would be insufficient for studying breathing-related immune conditions; air quality, for example, can vary enormously within a relatively short distance, and must be monitored at the very least at a street level. While several solutions have been proposed to infer the locations of tweets, these algorithms underperform when applied in the real world.^9^

An alternative approach is to use self-reporting methods. We suggest that the Experience Sampling Method (ESM), which has been widely used in a variety of fields,^10^ is well suited to collect allergy symptoms, due to its ecological validity and robustness against memory bias.^11^ In a nutshell, participants of ESM studies have to report on a particular issue, at a predetermined interval of time, and/or when triggered by a specific condition or situation. In this paper we address the question of whether ESM is a reliable method for capturing respiratory allergy symptoms in the UK. We established the #BritainBreathing citizen science project, whereby we asked participants to submit their wellness and allergy symptoms via the Britain Breathing mobile application.

## METHODS

### Design

**Figure 1.**
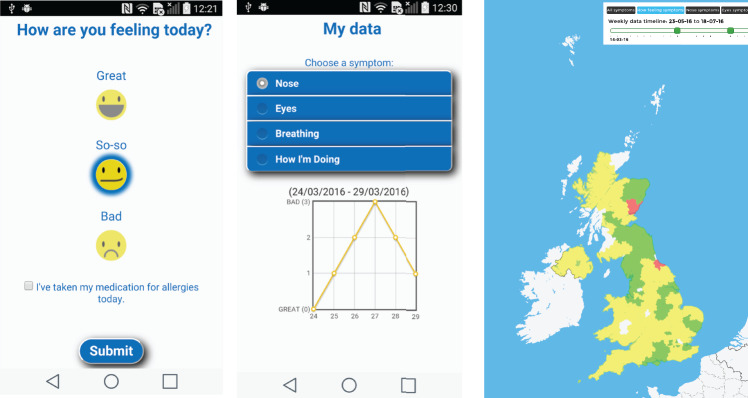
Screenshots of the Britain Breathing app showing well-being reporting screen (left), symptom evolution chart (center) and visualization of allergy incidence (right).

We ran a cross-sectional study with the Britain Breathing Android mobile app, which was co-designed by immunologists and allergy sufferers^12^ following the principles of participatory design.^13^ Once the app was installed, participants had to indicate their gender, year of birth, and whether they were suffering from any allergy, which was an optional field. The Data Sharing section contains the terms of consent, confidentiality, and anonymity of the data. Only those who gave their consent were able to unlock the symptom-reporting functionality, which implements the essential requirements for reporting allergy symptoms,^14^ including the minimum number of symptoms or variables to collect (nasal, eyes, breathing), the scale to be used (4-point scale: 0 = absent to 3 = severe), the type of data (ordinal), and the format of data (visual analog scale). Collecting information on whether users are taking medication was also considered relevant, as medications modulate the symptoms. This is indicated with a check-box at every submission (Figure 1, left), although we do not ask about the type of medication taken; therefore, we do not know if those who tick the box receive clinically recommended treatments. 

On the app, users report how they are feeling: the choices are “good,” “so-so,” and “bad” (Figure 1, left), which constitutes the wellness score on a scale of 0–2, where lower values indicate greater wellness. If symptoms are reported, details about their severity are recorded via 3 sliders (1 per symptom) on the above-mentioned 4-point scale. Every report has an associated timestamp and geographical coordinates that are automatically collected through the mobile phone’s clock and Global Positioning System (GPS), respectively.

Not only can users browse the evolution of their own symptoms on a line chart (Figure 1, center), but they can also explore allergy incidence on a widget, which is openly available on the Web and allows users to filter the data per symptom, wellness, and period (Figure 1, right). The Britain Breathing app lets individuals submit their reports at any time (ie, event-contingent sampling) and at intervals that are scheduled by participants through once-a-day alerts (ie, interval-contingent sampling).

### Participants

The app was released on March 18, 2016, via the Google Play store and data was collected until October 30, 2016. We publicized the Britain Breathing project through social media, blogs, websites, public engagement activities, and appearances at science festivals and on public television.

As we intended the dataset, which included participant location, to be openly available, we did not univocally identify participants, and instead used the combination of the *year of birth* and *gender* variables to approximate the number of participants in each postcode. This method is liable to under-report individuals of the same gender born in the same year (ie, all women born in 1966 reporting from Manchester postcodes are counted as one) and over-report individuals who submitted their symptoms from different postcodes, and therefore its advantages from the perspective of conserving participant anonymity are traded off against its ability to identify individuals. Overall, this method appeared to over-report by 1.9%, if we consider the number of downloads of the app as the expected value. This estimate suggests that the median age of participants was 41 years (SD = 14.48) and 51% of them were men.

## RESULTS

### App usage

**Figure 2.**
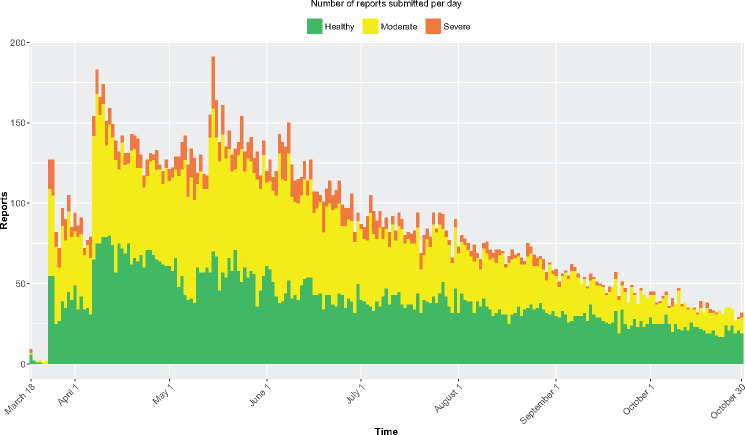
The number of reported entries per day and their severity.

At the end of the study (October 30, 2016) the Britain Breathing app had been downloaded 1530 times and 425 people had the app installed on their phones, which means the app had been uninstalled 1105 times. We collected 20 278 observations. Figure 2 shows the number of reports submitted per day. 

We hypothesize that the seasonal effect is relatively strong, as the time when people reported feeling worst (weeks 18–25 of the year in Figure 2: May 2–June 20, 2016) was the period during which 35% of the observations were reported. Following this, as the average lack of well-being and symptom severity began to go down, so did the number of reports. This is supported by the relative number of reports per well-being score: we observed a reduction of 23 percentage points in people feeling unwell (moderate + bad) when comparing June and October, which is corroborated by a positive correlation between the number of reports and average lack of wellness per week (*r* = 0.73, *P* < .05), with both variables going down. The proportion of reports submitted by those who were feeling *great* was 48% on average, reaching 55% and 62% in the months of lower incidence, September and October, respectively, which suggests good retention of participants.

### Coverage

We received at least one report from 118 of the 124 postcode areas in the UK, which accounts for 95% of all postcode areas. Average reports per postcode were 167 (min = 1, max = 613, SD = 156). At least one report was received from 43% of postcodes during every month of the study (8 months), and at least one report from 69% of postcodes over a 7-month period.

### Validity

As a way of cross-checking the validity of our data, we compared it with antihistamine prescription data (corresponding to British National Formulary section 3.4.1^15^) for seasonal allergies over the same period. There is a strong correlation (*r* = 0.93, *P* < .003) between the median lack of wellness and the number of antihistamines prescribed by general practitioners during the April–October 2016 period.^16^

### Symptoms

**Figure 3.**
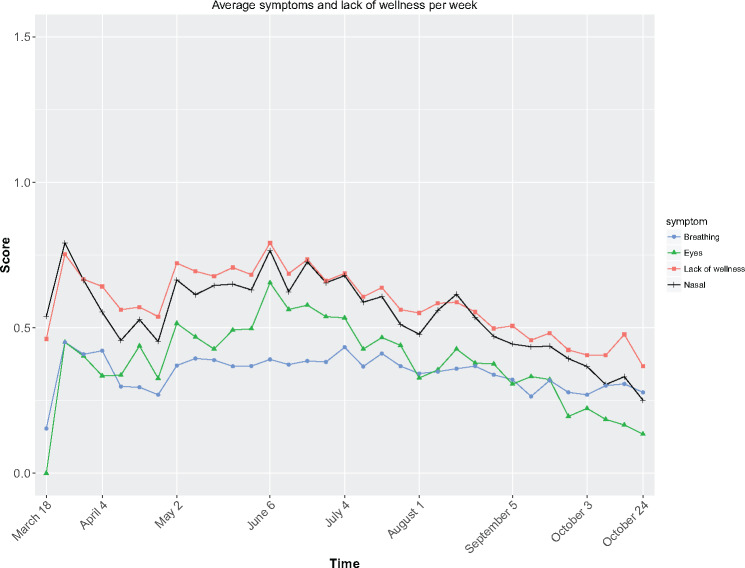
Average wellness and symptoms per week collected through the Britain Breathing app for breathing, eyes, and nasal symptoms as well as for wellness. Note that symptoms are on a 4-point scale (0 = absent and 3 = severe), while wellness is on a 3-point scale (0 = good, 1 = so-so, 2 = bad).

Our data suggest that, in reporting allergy symptoms, eye and nasal symptoms act as an indicator of overall lack of wellness (Figure 3). 

Spearman correlations confirmed that nasal symptoms had the strongest correlation with wellness, *r* = 0.75, followed by eye symptoms, *r* = 0.62, and breathing symptoms, *r* = 0.57; all symptoms, *P* < .0001. This means that a blocked or a runny nose had a greater impact on well-being than itchy or watery eyes. Indeed, these 3 symptoms explain 64% of the variance shown by well-being, *R*^2^ = 0.64, *F*(3,19746) = 11600, *P* < .0001.

People taking medication for their symptoms accounted for 51% of the entries. Those who took medication reported feeling worse than those who did not, with average values of 0.73 vs 0.47, respectively (note that higher values indicate lower well-being). This is confirmed by a Mann-Whitney U test: U = 1068, *Z* = −6.7, *P* < .0001, which suggests a relationship between drug taking and well-being. Although the cause of the relationship cannot be confirmed, it is possible that people with more severe symptoms are more likely to take medication. While this finding might be obvious, it provides further evidence on the reliability of the ESM.

## DISCUSSION

Our study indicates that ESM, delivered via a mobile device, is a reliable method of collecting data about respiratory allergy symptoms within a country. This is supported by the strong relationship between the reported well-being of the participants and the number of antihistamines prescribed, and from the wide geographical distribution of the reports that were collected during the period in which the study ran. We see 2 peaks of rhinitis symptoms in April, which is likely due to tree pollens, and a second peak in June, which is likely due to grass pollens, which are high at that time.^17^ The dataset generated during this study has already provided new insights. For instance, we found nasal symptoms to be most strongly related to well-being, and those who took medication reported feeling worse. In the future, it will provide a basis for investigating the relationship between allergy incidence and other factors, such as air quality and weather.

We acknowledge the known limitations of the ESM, including social desirability and self-selection biases, quality of the data, and attrition.^11^ In order to reduce the risks to validity, symptoms should not be treated as isolated variables, as they will be impacted by interactions of allergens with a range of triggers, including temperature and humidity, location (indoors/outdoors), and time of day. Some interactions, such as pollution and climate, could act to make allergens more immunogenic and stimulatory to the immune system, which suggests that we might need to include additional datasets to fully understand the data. As a starting point, with good location and symptom data, there is a good opportunity to characterize confounding variables that can impact symptoms. Attrition is an issue of the ESM, and the observed decline in reports over time (see Figure 2) is a well-documented pattern in citizen science projects,^18^ although it should be noted that the decline in this study occurred at a much lower rate than has been observed in other projects^19^ and dropouts do not have a significant effect on the quality of reported data.^20^ Attrition may also be impacted by the seasonality of allergy incidence; the likelihood is that it is a combination of both of these factors. Consequently, within the scope of this work and with the collected dataset, we can safely say that the benefits of the ESM outweigh its limitations.

## CONCLUSION

We provide evidence of the reliability of the ESM for collecting the first dataset of seasonal allergy symptoms (and their severity) with associated timestamps and geographic coordinates. This dataset and others generated by this method will be instrumental in understanding the causes of allergies.

## FUNDING

#BritainBreathing has received funding from the following organizations and grant schemes: Biotechnology and Biological Sciences Research Council Activating Impact award; British Society for Immunology; Medical Research Council award (MR/K006665/1), funded via the Health eResearch Centre; and Wellcome Trust Institutional Strategic Support Fund (105610/Z/14/Z).

## ETHICS

The School of Computer Science Ethics Committee approved this project, reference number CS 250.

## COMPETING INTERESTS

None.

## DATA SHARING

Data will be available at the Britain Breathing website: http://britainbreathing.org/.

## CONTRIBUTION

AB and SC developed the idea of the project. AB, SC, LH, CJ, and MV devised and participated in the workshops. SC and LH took care of all aspects of public involvement. MV and LH developed the prototypes after workshops. WV developed the mobile application. MV analyzed the data. MV and CJ wrote the manuscript. AB, SC, and LH critically edited the manuscript. All authors approved the manuscript.
